# Association of Serum Retinol Binding Protein 4 with Atherogenic Dyslipidemia in Morbid Obese Patients

**DOI:** 10.1371/journal.pone.0078670

**Published:** 2013-11-04

**Authors:** Milagros Rocha, Celia Bañuls, Lorena Bellod, Susana Rovira-Llopis, Carlos Morillas, Eva Solá, Víctor M. Víctor, Antonio Hernández-Mijares

**Affiliations:** 1 Service of Endocrinology, University Hospital Dr. Peset, Valencia, Spain; 2 Foundation for the Promotion of Healthcare and Biomedical Research in the Valencian Community (FISABIO), Valencia, Spain; 3 CIBER CB06/04/0071 Research Group. CIBER Hepatic and Digestive Diseases, Faculty of Medicine, University of Valencia, Valencia, Spain; 4 Institute of Health Research INCLIVA, Valencia, Spain; 5 Department of Medicine, Faculty of Medicine, University of Valencia, Valencia, Spain; 6 Department of Physiology, Faculty of Medicine, University of Valencia, Valencia, Spain; INSERM/UMR 1048, France

## Abstract

Retinol binding protein 4 (RBP4) is an adipokine that may contribute to the development of insulin resistance. However, how this adipokine is affected and its possible involvement in lipid metabolism in obese patients with varying degrees of insulin resistance is yet to be determined. A total of 299 middle-aged morbid obese patients (BMI>40 kg/m^2^) were divided in euglycemic, metabolic syndrome or type 2 diabetic. Anthropometric measurements, biochemical variables and systemic RBP4 levels were determined. RBP4 levels were significantly higher in patients with metabolic syndrome and type 2 diabetes than in euglycemic subjects (42.9±14.6; 42.3±17.0 and 37.4±11.7 µg/ml, respectively) and correlated with triglycerides but not with those of HOMA-IR in the whole population. The multivariate regression model revealed that triglycerides were the strongest predictor of systemic RBP4 levels. Analysis of lipoprotein subfractions in a subpopulation of 80 subjects showed an altered profile of insulin resistant states characterized by higher VLDL, sdLDL and small HDL percentages and lower large HDL percentage. Although RBP4 levels correlated significantly with LDL particle size and small HDL percentage, the latter parameter was independently associated only with RBP4. Our study reveals that systemic RBP4 levels could play an important role in lipid metabolism in morbid obesity, increasing triglyceride levels and contributing to the formation of small HDL.

## Introduction

Obesity is a prevalent condition characterized by the excessive accumulation and storage of fat in adipose tissue. It is a major cause of insulin resistance, which is implicated in the development of metabolic syndrome (MetS) and type 2 diabetes (T2D). These pathologies are related with the development and progression of atherosclerosis, one of the leading causes of morbidity and mortality in industrialized countries [1].

Dyslipidemia is a known risk factor for atherosclerosis and is commonly linked to insulin resistance and, hence, to obesity, MetS and T2D [2]. The two major quantitative lipid abnormalities in these patients are increased triglyceride levels and low high-density lipoprotein (HDL) levels. When these parameters are associated with a higher proportion of small and dense low-density lipoprotein particles (sdLDL), atherogenic dyslipidemia is diagnosed. This condition has emerged as an important marker of the increased cardiovascular disease risk observed in patients with obesity, MetS, insulin resistance and T2D [3]. HDL does not represent a sum of identical particles, but rather a collection of discrete subfractions that differ with respect to their physicochemical properties; namely size, density, composition and charge. In common metabolic diseases, such as T2D and MetS, it seems that HDL atherogenicity increases as particle size decreases, and that small HDL particles are more atherogenic than large HDL [4].

Retinol binding protein 4 (RBP4), secreted primarily by the liver and adipocytes, is a recently identified adipokine [5] apparently linked to obesity and its comorbilities in humans, especially insulin resistance, T2D, and certain components of MetS. However, it is a matter of controversy whether RBP4 levels in obesity are determined by insulin resistance. Circulating RBP4 levels have been shown to rise and to positively correlate with body mass index (BMI) [5], [6] and to be associated with insulin resistance [5]–[8], while there have been contrasting reports of similar RBP4 levels regardless of BMI and no significant correlation between RPB4 and insulin resistance [9]–[12].

In addition, growing evidence suggests that RBP4 plays a role in lipid metabolism to an even greater extent than insulin resistance. In fact, many human studies have found a strong relationship between RBP4 and triglycerides, some finding an association with insulin resistance [6], [13] and others failing to do so [14]-[16]. Indeed a role for RBP4 in the catabolism of very low-density lipoprotein (VLDL) [14] and the formation of sdLDL has been suggested [17], [18], though the implication of RBP4 in components of atherogenic dyslipidemia has been studied very little.

Thus, in order to gain further insight into the relation between RBP4, insulin resistance and lipid metabolism, we studied a large cohort of obese subjects with different degrees of insulin resistance. The following aspects were explored: 1) the potential association of serum RBP4 levels with insulin resistance; 2) metabolic parameters as independent predictors of RBP4; and 3) the involvement of RBP4 in contributing to the atherogenicity of both LDL and HDL particles.

## Materials and Methods

Our study population was composed of patients with a BMI >40 kg/m^2^ who were referred to the Outpatient's Department of the Endocrinology and Nutrition Service at the University Hospital Dr. Peset in Valencia to be treated for obesity. The study was conducted according to the guidelines laid down in the Declaration of Helsinki, and all procedures involving human subjects were approved by the Ethics Committee of the University Hospital Dr Peset. Written informed consent was obtained from all the participants.

The MetS was based on the updated National Cholesterol Education Program Adult Treatment Panel III criteria [19], by which at least three of the following parameters must be present: 1) waist circumference of 102 cm or greater in men or 88 cm or greater in women; 2) triglyceride levels of 150 mg/dl or greater; 3) HDLc under 40 mg/dl in men or under 50 mg/dl in women; 4) blood pressure 130/85 mm Hg or greater or current use of antihypertensive medication; and 5) fasting plasma glucose levels of 100 mg/dl or greater, or previously diagnosed T2D, or being on oral antidiabetic agents or insulin. Type 2 diabetes was defined according to the American Diabetes Association [20]: fasting glycaemia ≥126 mg/dl on at least two occasions, or glycaemia 2 h after 75 g glucose oral load of ≥200 mg/dl, or glycated hemoglobin ≥6.5%.

Exclusion criteria were pregnancy or lactation, recent change of oral contraceptive formulation, severe disease, history of cardiovascular disease or chronic inflammatory disease, pharmacological treatment for diabetes-antidiabetic agents or insulin-, secondary obesity (hypothyroidism, Cushing's syndrome), and pharmacological treatment for dyslipidemia or hypertension. According to these criteria, subjects were divided into three groups: euglycemic without MetS (Euglycemic group); metabolic syndrome patients without T2D (MetS group) and metabolic syndrome patients with newly diagnosed type 2 diabetes (Type 2 diabetic group).

Anthropometrical parameters were evaluated as follows: weight was determined using electronic scales with an approximation of 0.1 kg and a capacity of up to 200 kg; height was measured with a stadiometer with an approximation of 0.5 cm; BMI was calculated by dividing the weight in kilograms by the square of the height in meters; blood pressure was measured twice consecutively using a mercury sphygmomanometer; and waist and hip circumference was measured at the natural indentation between the 10^th^ rib and the iliac crest in the former case and at the height of the major trochanter using a metric tape in the latter, with approximations of 0.5 cm in both cases.

Venous blood samples were collected from patients after 12-h overnight fasting and placed in a BD Vacutainer® Rapid Serum Tube (Becton Dickinson, New Jersey, USA). To separate serum from blood cells, samples were centrifuged at 2,000g for 15 min at 4°C following 5-min clotting. Freshly separated serum was employed for determination of lipid profile and hydrocarbonated parameters, and the remaining aliquots of serum were stored at −80°C until they were assessed for circulating levels of RBP4 and lipoprotein subfractions.

Glucose, total cholesterol and triglycerides were measured by means of enzymatic assays and HDLc using a direct method and a Beckman LX-20 autoanalyzer (Beckman Coulter, La Brea, CA, USA). The intraseries coefficient of variation was <3.5% for all these determinations. LDLc was calculated using Friedewald's formula for values of triglycerides below 300 mg/dl. Apolipoproteins (Apo) AI and B were determined by immunonephelometry (Dade Behring BNII, Marburg, Germany) (intra-assay coefficient of variation <5.5%) and insulin was determined by immunochemiluminescence with an Immulite analyzer (DPC, Los Angeles, USA) (intra-assay coefficient of variation <4%). Insulin resistance was estimated by the homeostasis model assessment of insulin resistance (HOMA-IR) index (fasting insulin (µIU/ml) x fasting glucose (mg/dl) /405). Serum RBP4 concentrations were measured by nephelometry (Dade Behring, Marburg, Germany). This method has a sensitivity of 0.1 µg/ml. The intra- and interassay coefficients of variation were 3.1% and 2.2%, respectively.

LDL and HDL subfractions were separated using the Quantimetrix Lipoprint system (Redondo Beach, CA,USA) [21] and then identified and quantified using a computerized method developed for the Quantimetrix Lipoprint system and NIH image program version 1.62 (Bethesda, MD, USA) for research purposes. The Liposure® (Quantimetrix Corporation, Redondo Beach, CA, USA) was used for quality control. VLDL, 3 intermediate-density lipoprotein (IDL), 7 LDL and 10 HDL subclasses were quantified. The LDL electrophoretic profile allows 3 patterns to be defined: pattern A/ large and buoyant LDL (cut-off size more than 268 Å); intermediate pattern (cut-off size more than 265 and equal to or less than 268 Å); and pattern B/sdLDL (cut-off size less than or equal to 265 Å). At the same time, HDL particles were classified as large (subfractions 1–3), intermediate (subfractions 4–7) or small (subfractions 8–10).

The statistics program SPSS version 17.0 was employed for statistical analysis. Continuous variables were expressed as mean and standard deviation (SD) or as median and 25^th^ and 75^th^ percentiles for parametric and non-parametric data, respectively. Qualitative data were expressed as percentages. Data with normal distribution were compared with a one-way analysis of variance (ANOVA) and those without normal distribution were compared with a Kruskal-Wallis, followed in each case by a post hoc test (Student-Newman-Keuls or Dunn's multiple comparison, respectively) or an independent T-test or Mann-Whitney U when the dependent variable was not normally distributed. The Chi-Square test was used to compare proportions. Spearman's correlation coefficient was employed to measure the strength of the association between RBP4 and anthropometric and metabolic parameters. In the multivariable regression models, the relationship between two or more explanatory variables (independent variables) and a response variable (dependent variable) was evaluated by fitting a linear equation to the data obtained. After identification of independent variables, these variables were introduced as covariates in a univariate general linear model. All the tests had a confidence interval of 95% and differences were considered significant when p<0.05.

## Results

This study analysed a total of 299 patients (102 men and 197 women). When the distribution of RBP4 was assessed according to gender, we found it to be significantly higher in men than in women (45.1±15.6 µg/ml versus 39.3±13.4 µg/ml, p<0.0001, respectively).

Anthropometric and biochemical parameters for obese patients with MetS, T2D and euglycemia are shown in Table 1. Patients with MetS and T2D had significantly higher systolic and diastolic blood pressure, Apo B and insulin, and lower HDLc and Apo AI than obese euglycemic subjects but a similar level in both groups. Significant differences were found between the three groups in age, triglyceride levels and hydrocarbonated metabolism parameters (glucose and HOMA-IR) (p<0.001 for all variables), whereas waist circumference differed significantly only between T2D and euglycemic subjects (greater in the former).

**Table 1:** Anthropometric parameters and biochemical variables in obese patients with different degrees of insulin resistance.

	Euglycemia	MetS	Type 2 diabetes	p-value
n	84	164	51	—
Men (%)	21 (25.0)	61 (37.2)	20 (39.2)	0.117
Age (years)	36.8±11.9 ^a^	41.4±11.2 ^b^	46.9±9.7 ^c^	<0.001
BMI (kg/cm^2^)	46.7±6.1	47.5±6.3	48.1±6.4	0.444
Waist circumference (cm)	127.6±15.2 ^a^	131.6±13.8 ^a,b^	135.4±12.1 ^b^	0.012
Systolic BP (mm Hg)	118.2±11.4 ^a^	137.4±19.8 ^b^	135.9±23.0 ^b^	<0.001
Diastolic BP (mm Hg)	71.8±8.4 ^a^	83.9±14.3 ^b^	79.5±15.9 ^b^	<0.001
Total cholesterol (mg/dl)	185.0±40.4	187.7±36.8	198.2±44.1	0.152
HDLc (mg/dl)	45.9±10.9 ^a^	37.2±7.9 ^b^	34.6±7.2 ^b^	<0.001
LDLc (mg/dl)	119.1±35.4	122.0±31.6	128.3±31.2	0.303
Triglycerides (mg/dl)	101 (73, 126) ^a^	137 (101, 179) ^b^	153 (111, 202) ^c^	<0.001
Apo AI (mg/dl)	134.5±25.7 ^a^	125.1±20.6 ^b^	122.3±19.6 ^b^	0.002
Apo B (mg/dl)	97.3±26.7 ^a^	106.9±28.4 ^b^	114.3±28.2 ^b^	0.003
Glucose (mg/dl)	86.4±9.9 ^a^	96.6±15.7 ^b^	157.7±40.2 ^c^	<0.001
Insulin (µU/ml)	13.9±7.7 ^a^	18.2±10.3 ^b^	18.1±11.0 ^b^	0.008
HOMA-IR index	2.95±1.64 ^a^	4.40±2.65 ^b^	6.93±4.17 ^c^	<0.001
RBP4 (µg/ml)	37.4±11.7 ^a^	42.9±14.6 ^b^	42.3±17.0 ^b^	0.016

Data are expressed as mean ± standard deviation for parametric data or as median (25^th^ and 75^th^ percentiles) for non-parametric data. Values with different superscripts (a,b,c) indicate that the differences among groups are significant (p<0.05) when compared by means of one-way ANOVA or Kruskal-Wallis for data with normal or without normal distribution, respectively, followed by a post hoc test.

Abbreviations: MetS, metabolic syndrome; BMI, body mass index, BP, blood pressure; RBP4, retinol binding protein-4, Apo, apolipoprotein; HOMA-IR, homeostasis model assessment of insulin resistance.

In contrast, no differences were observed among the patient groups in relation to BMI (p = 0.444), total cholesterol (p = 0.152) or LDLc (p = 0.303). Serum levels of RBP4 were higher in MetS and T2D patients than in euglycemic subjects and were similar for the two insulin-resistant states.

**Figure 1:**
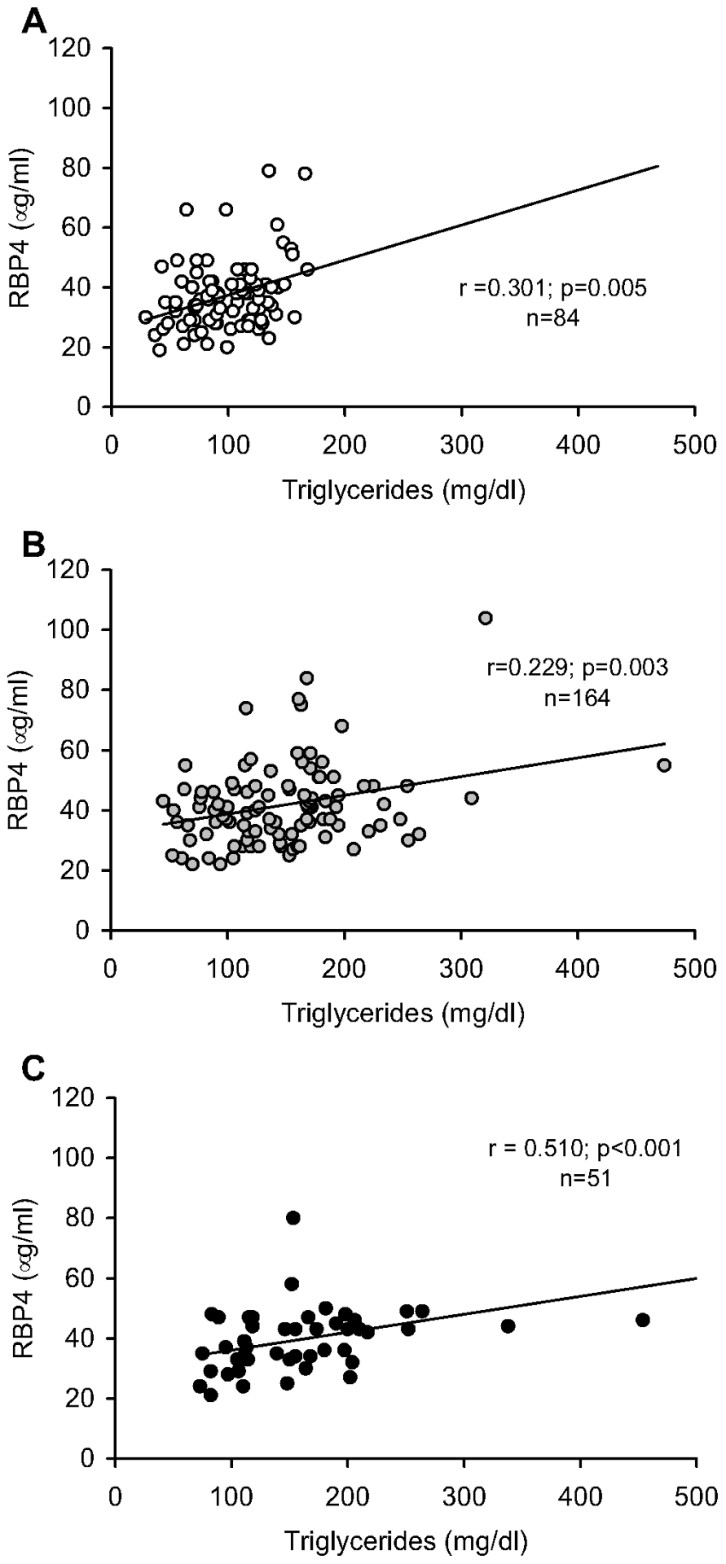
Bivariate correlation between serum RBP4 levels and triglycerides in obese patients. A: Euglycemic patients without metabolic syndrome; B: metabolic syndrome patients (MetS); C: type 2 diabetic patients (T2D). All correlation coefficients (r) are given as calculated by Spearman's correlation.

Spearman correlation analysis demonstrated the strongest correlation between RBP4 and triglycerides (r = 0.335; p<0.001), which was observed in the whole population. This positive correlation was detected for each of the populations studied –euglycemic, MetS and T2D- as shown in Figure 1. Likewise, RBP4 correlated with waist circumference (r = 0.131; p = 0.033), systolic and diastolic blood pressure (r = 0.224 and r = 0.165, respectively, p<0.01), total cholesterol (r = 0.229; p<0.001), LDLc (r = 0.147; p = 0.012) and Apo B (r = 0.270; p<0.001). In contrast, significant bivariate associations were not detected between RBP4 and BMI (r = −0.075; p = 0.195), HDLc (r = −0.050; p = 0.296) fasting glucose (r = −0.005; p = 0.927), insulin (r = −0.058; p = 0.368) or HOMA-IR (r = −0.015; p = 0.809).

**Figure 2:**
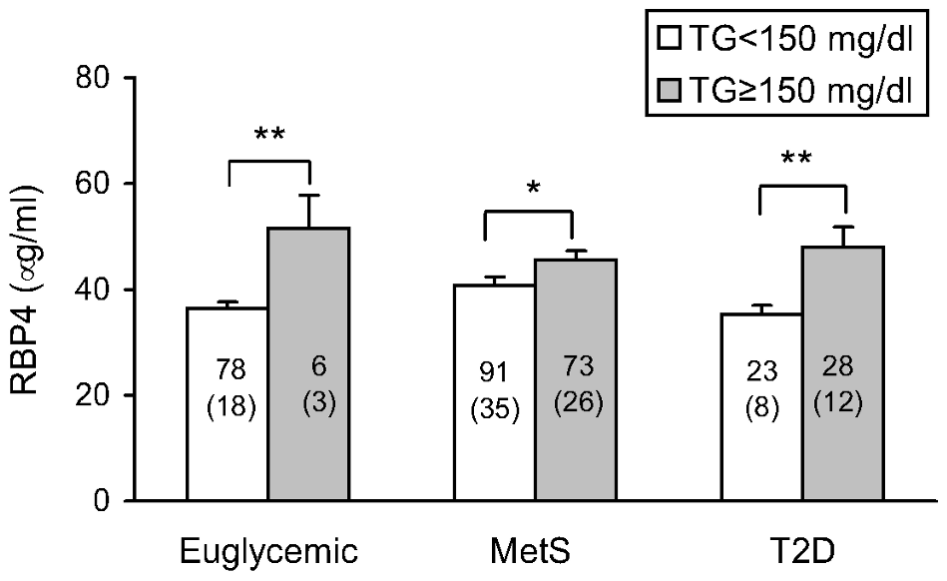
Serum RBP4 levels in euglycemic, metabolic syndrome and type 2 diabetic patients according to optimal or high triglyceride levels. Data are expressed as mean+SEM. Sample size is indicated inside the column and the number of men is indicated in brackets. *p<0.05, **p<0.01 when compared by unpaired T-test optimal versus high triglycerides. A Pearson Chi square test revealed no differences in sex distribution between optimal and high triglyceride levels. Abbreviations: MetS: metabolic syndrome patients; T2D: type 2 diabetic patients.

In multivariable regression models, the association of RBP4 with sex, age, BMI, waist circumference, total cholesterol, HDLc, LDLc, triglycerides, glucose, insulin and HOMA-IR was evaluated in each case as a potential independent predictor using the stepwise method. Our results showed that total cholesterol, triglycerides and gender were independently associated with RBP4 (Table 2). To further investigate how these variables influence RBP4 in pathologies characterized by varying degrees of insulin resistance, we adjusted RBP4 for independent variables. Differences in serum RBP4 levels among the three groups –euglycemic, MetS and T2D- disappeared after adjustment for triglyceride levels (p = 0.362). Finally, we evaluated whether triglycerides were responsible for the differences in RBP4 among our patients. In fact, when we divided the population according to normal (<150 mg/dl) or high triglyceride levels (≥150 md/dl) and performed a one-way ANOVA we found no differences among the groups. However, we did observe a significant increase in serum RBP4 levels in patients with high levels of triglycerides independently of their degree of insulin resistance (Figure 2) and gender. Indeed, when we calculated the sex ratio between high and normal triglyceride levels, we found normal triglyceride levels in 85.7% of men and 95.2% of women (p = 0.142) in the euglycemic group, 57.3% of men and 54.4% of women (p = 0.708) in the MetS group, and 40.0% of men and 48.4% of women (p = 0.557) in the T2D group.

**Table 2:** Stepwise multivariate regression model for anthropometric and biochemical variables and serum RBP4 levels as dependent variable (n = 299).

	Unstandardized coefficients		Standardized coefficients	p value
	B	SE	β	
Total cholesterol	0.060	0.020	0.167	0.023
Triglycerides	19.54	5.13	0.281	<0.001
Gender (women)	−5.06	1.83	−0.179	0.006
Multiple R square-adjusted			0.188	
R			0.445	
p			<0.001	

Triglyceride levels were a log-transformed variable.

Age, body mass index, waist circumference, low-density lipoprotein cholesterol, high-density lipoprotein cholesterol, glucose, insulin and homeostasis model assessment of insulin resistance were excluded from the model.

LDL and HDL subfractions were also analysed in a subpopulation of 80 subjects to determine the link between plasma RBP4 and lipoprotein metabolism. An altered lipoprotein profile -higher VLDL and sdLDL percentages and lower HDL percentage with a more proatherogenic profile- was associated with insulin-resistant state (Table 3). This was consistent with the reduction of estimated LDL particle size and a higher prevalence of the atherogenic LDL pattern. Interestingly, RBP4 significantly correlated with LDL particle size (r = −0.354, p<0.001) and small HDL (r = 0.425, p<0.001). To analyze whether this association was independent of triglycerides; a multivariable linear regression was performed in which LDLc, HDLc, triglycerides, glucose, insulin, HOMA-IR and RBP4 were included as independent variables (Table 4). The stepwise model showed that LDLc, HDLc and triglyceride levels were independent predictors of both LDL particle size and small HDL, although a specific role of RBP4 was determined (β = 0.214) in the latter case.

**Table 3:** Anthropometric, biochemical characteristics and lipoprotein subfractions in obese patients according to different degrees of insulin resistance.

	Euglycemia	MetS	Type 2 diabetes	p-value
n	21	31	28	
Men (%)	7 (33.3)	7 (22.6)	14 (50)	0.086
BMI (kg/cm^2^)	46.5±5.8	45.1±5.0	46.7±6.8	0.535
Biochemical parameters				
Total cholesterol (mg/dl)	176.1±37.9	181.7±38.1	200.4±44.6	0.083
HDLc (mg/dl)	46.1±13.4 ^a^	38.0±8.5 ^b^	36.4±9.2 ^b^	0.003
LDLc (mg/dl)	109.9±32.8	115.4±31.7	129.4±35.3	0.133
Triglycerides (mg/dl)	93.0 (73.0, 125.5) ^a^	127.0 (94.0, 182.0) ^b^	156.0 (114.3, 213.8) ^c^	<0.001
RBP4 (µg/ml)	32.5±7.0 ^a^	39.6±6.4 ^b^	41.6±14.3 ^b^	0.034
Lipoprotein subfractions (%)				
VLDL	17.7±4.2 ^a^	22.3±3.2 ^b^	19.8±3.6 ^c^	<0.001
IDL	31.0±5.8	29.8±5.8	28.2±2.8	0.132
Large LDL	23.9±8.0 ^a^	24.9±5.4 ^a^	31.2±3.9 ^b^	<0.001
Small dense LDL	0.79±1.01 ^a^	1.83±2.58 ^a,b^	2.89±3.22 ^b^	0.020
HDL	26.4±5.6 ^a^	21.0±4.3 ^b^	18.7±4.2 ^b^	<0.001
LDL electrophoretic profile				
A LDL pattern (%)	90.5 ^a^	80.6 ^b^	71.4 ^c^	<0.05
Intermediate LDL pattern (%)	9.5	9.7	3.6	>0.05
B LDL pattern (%)	0 ^a^	9.7 ^b^	25.0 ^c^	<0.05
LDL size (Å)	272.6±2.5 ^a^	270.3±3.8 ^b^	268.9±4.5 ^b^	0.005
HDL electrophoretic profile (%)				
Large HDL (1–3)	31.3±7.2 ^a^	24.9±5.7 ^b^	21.4±6.6 ^c^	<0.001
Intermediate HDL (4–7)	52.2±4.8	52.7±4.0	51.8±3.5	0.684
Small HDL (8–10)	16.4±4.2 ^a^	22.4±7.1 ^b^	26.8±8.0 ^c^	<0.001

Data are expressed as mean ± standard deviation for parametric data or as median (25^th^ and 75^th^ percentiles) for non-parametric data. Values with different superscripts (a,b,c) indicate that the differences among groups are significant (p<0.05) when compared by means of one-way ANOVA or Kruskal-Wallis for data with normal or without normal distribution, respectively, followed by a post hoc test.

Abbreviations: MetS, metabolic syndrome; BMI, body mass index, RBP4, retinol binding protein-4; VLDL: very low-density lipoprotein; IDL: intermediate-density lipoprotein; LDL: low-density lipoprotein; HDL: high-density lipoprotein.

**Table 4:** Stepwise multivariate regression model for biochemical variables and LDL particle size and small HDL as dependent variables.

Dependent variable	Independent variables	Unstandardized coefficients		Standardized coefficients	p-value
		B	SE	β	
LDL size	LDLc	−0.035	0.010	−0.361	<0.001
	HDLc	0.075	0.032	0.233	0.021
	Triglycerides	−7.24	2.13	−0.372	<0.001
Multiple R square adjusted			0.448		
R			0.688		
p			<0.001		
Small HDL	LDLc	0.071	0.023	0.311	0.003
	HDLc	−0.219	0.075	−0.291	0.005
	Triglycerides	15.9	4.97	0.347	0.002
	RBP4	0.136	0.061	0.214	0.029
Multiple R square adjusted			0.489		
R			0.721		
p			<0.001		

Triglycerides were a log-transformed variable.

All variables (total cholesterol, LDLc, HDLc, triglycerides, glucose, insulin, HOMA-IR and RBP4) that significantly correlated with LDL particle size in the Spearman's analysis were taken to be independent. Total cholesterol, glucose, insulin, HOMA-IR and RBP4 were excluded from the model since they were not significant predictors (p>0.05).

## Discussion

The current study provides evidence that serum RBP4 levels are higher in middle-aged morbid obese subjects with MetS and T2D than in euglycemic ones. We also show that triglycerides are the main independent predictor for determining systemic RBP4 levels, independently of the degree of insulin resistance. In addition, we demonstrate an independent association among small HDL percentage and LDLc, HDLc, triglycerides and RBP4, but not between LDL particle size and RBP4. Our data suggest a central role of this adipokine in the hypertriglyceridemia associated with insulin-resistant state and point to it as an independent predictor of the formation of small HDL.

In accordance with previous reports [10], [22], [23], serum RBP4 concentrations were found to be sexually dimorphic -higher in men than in women- in our study population. This has previously been reported for other adipokines and has been explained on the basis of varying fat amounts and the influence of sex hormones [24]. However, recent findings point to a specific role of RBP4 in women as an independent predictor of cardiovascular disease [25], [26], while a correlation has been reported between plasma RBP4 concentrations and cardiovascular risk factors, predominantly in young men [27].

In the present study we have observed a positive correlation between RBP4 and waist circumference –a subrogate marker of central obesity- which is in line with previous findings [28]. A direct relationship between waist circumference, triglycerides and RBP4 has recently been described by Lin et al [29]. However, we have found no correlation with BMI, and so high circulating levels of RBP4 in individuals with a high degree of obesity may not be merely a consequence of excess adipose tissue. It is possible that visceral fat is more important than subcutaneous fat and total body fat in determining circulating RBP4 levels [30], [31].

It has previously been shown that obesity, presence of MetS and T2D -all pathogenic conditions accompanying insulin resistance- are associated with increased serum RBP4 levels [6], [32], although some studies have found no association between RBP4 and insulin resistance [9], [10], [12], [15].

In this context, growing evidence suggests that RBP4 plays a more important role in lipid metabolism than in insulin resistance. Several studies in humans have shown that RBP4 levels are positively correlated with those of triglycerides but not with insulin resistance [14], [15], [33], [34]. At the same time, other studies reporting a relationship between plasma levels of RBP4 and triglycerides have detected a strong link with insulin resistance and an independent association of triglyceride levels and insulin resistance with RBP4 [6], [13]. In accordance with previous findings, we have confirmed that plasma RBP4 levels are elevated in obese subjects with MetS and T2D but do not correlate with insulin resistance, but rather with triglycerides. In addition, triglycerides were independently associated with RBP4 in our patients. In fact, when we adjusted RBP4 levels for triglycerides, no significant differences were found among euglycemic, MetS and diabetic patients. In line with this, when the population was stratified as normal or hypertriglyceridemic, those with higher levels of triglycerides had the highest levels of RBP4, independently of their degree of insulin resistance. Taken as a whole, these data suggest that RBP4 may play a role in the pathophysiology of hypertriglyceridemia in insulin-resistant states. Given the basic function of RBP4 as a retinol binding protein, it is reasonable to speculate that RBP4 serves as a link between retinol metabolism and activation of nuclear receptors and may be implicated in the regulation of lipid homeostasis. Thus, retinoids and retinol-binding proteins can modulate lipid activities, such as the expression of several genes involved in hepatic and intestinal triglyceride production and secretion, the hepatic production of VLDL, and the regulation of ApoC-III production –a protein that delays the catabolism of VLDL particles- and β-oxidation [35]. In fact, Verges et al. have recently demonstrated that RBP4 is involved in VLDL catabolism [14].

The possible effect of this adipokine on lipoprotein subfractions has been the focus of little research until now, but our findings suggest a strong association between RBP4 and triglycerides and indicate that RBP4 is not directly involved in determining LDL particle size, despite the negative correlation between the two. In a multiple linear regression, the independent predictors of LDL particle size included LDLc, HDLc and triglycerides, but not RBP4. This finding contrasts with the results of two previous studies in which RBP4 was independently associated with sdLDL [17], [18], although it is worth noting that the studies in question were carried out in Asian populations and did not include an analysis of triglycerides. Strikingly, in our population, RBP4 was found to be an independent predictor of small HDL fraction in addition to the abovementioned lipid variables. As far as we know, this is the first time an association between small HDL fraction and RBP4 has been reported.

Patients with insulin resistance exhibit an increased flux of free fatty acids to the liver that increases the cytosolic triglyceride storage pool and promotes VLDL production. In the presence of increased plasma levels of VLDL and normal activity of cholesterol ester transfer protein (CETP), VLDL triglycerides can be exchanged for LDL- and HDL cholesterol. This exchange produces LDL particles enriched in triglycerides, which are rapidly lipolyzed by hepatic lipase, resulting in smaller, denser particles. Numerous studies have demonstrated that the predominance of sdLDL particles (even though they can carry the same total cholesterol content) correlates with the progression of atherosclerosis and earlier and more severe cardiovascular disease [36]–[38]. Triglyceride-rich HDL particles are also smaller and can be modified in further ways, including hydrolysis of their triglycerides by hepatic lipase and reduced cholesterol efflux from cells, which leads to lower concentrations and dysfunctional HDLc [39], [40]. The dysfunctionality of HDL has been associated with complications caused by obesity, such as inflammation. The protective capacity of HDL can be undermined through a variety of mechanisms, including changes in its composition, loss of cholesterol acceptor activity, and decreased hepatic processing and secretion of cholesterol [41], [42]. Recently, elevated RBP4 levels have been reported to directly contribute to endothelial inflammation through a mechanism involving the activation of NADPH oxidase and NF-κB, which promotes the stimulation of the expression of proinflammatory molecules involved in leukocyte recruitment and adherence to the endothelium (e.g. cell adhesion molecules and IL-6) [43]. Thus, RBP4 may regulate lipid homeostasis through a classic mechanism of action (e.g. as a carrier of retinoids, or activation of nuclear receptors). On the other hand, RBP4 could induce inflammation though a novel mechanism responsible for a reduction in the size of HDL and a loss of its functionality. RBP4 has been shown to alter lipid profile in several clinical trials with fenretinide, a synthetic retinoid designed for cancer therapy that increases renal clearance of RBP4. Lazzeroni and colleagues reported a statistically significant increase in HDLc between fenretinide- and placebo-treated post-menopausal healthy women [44], in line with previous results obtained in metastatic breast cancer patients [45]. Moreover, fenretinide prevented the increase in triglyceride levels associated with augmented HOMA in premenopausal women with a high risk of developing breast cancer [46].

Therefore, our results provide further evidence that RBP4 is involved in different stages of the atherosclerotic process; on the one hand, it may be partially responsible for the enhancement of triglyceride levels, which, in turn, promotes the production of VLDL. On the other hand, it seems to be implicated in the formation of small HDL.

We should point out that our study has several limitations. First, causality could not be assessed due to the cross-sectional nature. Therefore, a longitudinal study needs to be performed in the future in order to explore further the independent association of triglycerides, formation of small HDL and RBP4 levels. Second, our results cannot be applied to the general human population, since our population was exclusively Caucasian.

To summarise, circulating RBP4 may play a major role in lipid metabolism in morbid obesity in which it is associated with an increase in triglyceride levels and contributes to the formation of small HDL, independently of insulin-resistant state. These findings are in accordance with the premise that systemic RBP4 levels promotes the development of atherosclerosis.
