# Correction: Association of Serum Retinol Binding Protein 4 with Atherogenic Dyslipidemia in Morbid Obese Patients

**DOI:** 10.1371/annotation/4ee4f5f7-519f-402c-8ed2-dff0cc6c5e48

**Published:** 2013-12-13

**Authors:** Milagros Rocha, Celia Bañuls, Lorena Bellod, Susana Rovira-Llopis, Carlos Morillas, Eva Solá, Víctor M. Víctor, Antonio Hernández-Mijares

There is an error in the units labeled in the Y-axis of Figure 1 and Figure 2. The correct figure label is: RBP4(µg/ml) 

